# Predicting vehicle travel time on city streets for trip preplanning and predicting heavy traffic for proactive control of street congestion

**DOI:** 10.1038/s41598-024-61379-7

**Published:** 2024-05-07

**Authors:** Samer Nofal

**Affiliations:** Department of Computer Science, German Jordanian University, Amman, Jordan

**Keywords:** Traffic prediction, Traffic control, Time series traffic data, Machine learning, Civil engineering, Information technology

## Abstract

We investigate if the vehicle travel time after 6 h on a given street can be predicted, provided the hourly vehicle travel time on the street in the last 19 h. Likewise, we examine if the traffic status (i.e., low, mild, or high) after 6 h on a given street can be predicted, provided the hourly traffic status of the street in the last 19 h. To pursue our objectives, we exploited historical hourly traffic data from Google Maps for a main street in the capital city of Jordan, Amman. We employ several machine learning algorithms to construct our predictive models: neural networks, gradient boosting, support vector machines, AdaBoost, and nearest neighbors. Our experimental results confirm our investigations positively, such that our models have an accuracy of around 98–99% in predicting vehicle travel time and traffic status on our study’s street for the target hour (i.e., after 6 h from a specific point in time). Moreover, given our time series traffic data and our constructed predictive models, we inspect the most critical indicators of street traffic status and vehicle travel time after 6 h on our study’s street. However, as we elaborate in the article, our predictive models do not agree on the degree of importance of our data features.

## Introduction

Street traffic congestion is a worldwide concern due to the substantial negative impact on society’s safety, economy, and environment^1–16^. Predicting traffic status can help with personal travel planning and support authorities for street proactive management, hence mitigating traffic congestion.

Map applications, such as Google Maps, are prevalent worldwide for personal planning of a trip starting *now* due to their ability to track the current traffic status through dynamic systems operating on users’ mobile devices. Nonetheless, suppose one inquires, for instance, on Google Maps about the vehicle travel time of a trip that starts after 6 h. In that case, Google Maps gives a loose estimate of the vehicle travel time by displaying to the user this message “typically *x* minutes to *y* minutes” where the difference between *x* and *y* is significant. Likewise, map applications fall short if traffic authorities want to estimate congestion for specific streets after 6 h.

Therefore, in this article, we investigate if the vehicle travel time after 6 h on a given street can be predicted, provided the hourly vehicle travel time on the given street in the last 19 h. Likewise, we examine if the traffic status (i.e., low, mild, or high) after 6 h on a given street can be predicted, provided the hourly traffic status of the street in the last 19 h. To pursue our objectives, we exploited historical hourly traffic data from Google Maps for a main street in the capital city of Jordan, Amman. We employ several machine learning algorithms to construct our predictive models: neural networks, gradient boosting, support vector machines, AdaBoost, and nearest neighbors. Our experimental results confirm our investigations positively, such that our models have an accuracy of around 98–99% in predicting vehicle travel time and traffic status on our study’s street for the target hour (i.e., after 6 h from a specific point in time). Moreover, given our time series traffic data and our constructed predictive models, we inspect the most critical indicators of street traffic status and vehicle travel time after 6 h on our study’s street. However, as we elaborate in the article, our predictive models do not agree on the degree of importance of our data features.

In the rest of this article, we discuss related work in “Related work”, elaborate on our methodology in “Methodology”, discuss our experimental results in “Experimental results”, and conclude the paper in “Conclusion”.

## Related work

In the literature, we find many studies on street traffic congestion prediction. In the following discussion, we highlight recently published related work, in which the interested reader may find further citations. Hence, the article^17^ discusses the prediction and modeling of traffic flow of human-driven vehicles at a signalized street intersection using an artificial neural network model. The result of^18^ proposes and evaluates the use of the Ising model for traffic congestion prediction. The paper of^19^ suggests a vision transformer approach for traffic congestion prediction on a city-wide scale. In^20,21^, the authors devise a traffic congestion prediction model based on a deep learning model. The work of^22^ integrates traffic science with representation learning for city-wide congestion prediction. In^23^, the authors use a multilayered deep neural network for traffic congestion prediction. The article of^24^ discusses utilizing a recurrent high-resolution network for large-scale traffic congestion prediction. In^25^, the authors implement a recurrent neural network for traffic congestion prediction. The work of^26^ applies a hybrid method combining swarm optimization and machine learning algorithms for traffic congestion prediction. The paper of^27^ presents a traffic congestion prediction model using seasonal auto-regressive integrated moving average and bidirectional long short-term memory for Internet of Things-enabled cities. In^28^, the authors tackle the problem of urban traffic congestion level prediction using a fusion-based graph convolutional network. The result of^29^ combines congestion speed-cycle patterns and a deep-learning neural network for short-term traffic speed predicting. In^30^, for traffic congestion prediction, the authors implement and evaluate four machine learning techniques: feed-forward neural networks, radial basis function neural networks, simple linear regression model, and polynomial linear regression model. In^31^, a data-driven model is constructed to predict urban street traffic congestion by using spatiotemporal characteristics of traffic zones’ traffic flow and utilizing convolutional long short-term memory and convolutional neural networks. The work of^32^ conducts a comparative analysis of street safety and prevention of world challenges in low-income and high-income countries. In^33^, the authors use dynamic people-flow and rainfall data and a transformer-based prediction model for traffic congestion prediction. The thesis of^34^ studies the prediction and mitigation of street traffic congestion based on machine learning. The work of^35^ develops a convolutional neural network and recurrent neural network-based algorithm for traffic congestion prediction. In^36^, the authors propose a congestion-aware traffic prediction system based on pipelined time variant feature selection. The dissertation of^37^ analyzes traffic congestion prediction and vehicle re-routing strategy using an image-based surveillance camera. The article of^38^ discusses traffic congestion mitigation by deceleration control with short-term velocity predicting. The paper of^39^ compares the performance of tree-based learning and support vector machines in traffic congestion prediction. The work of^40^ utilizes spatiotemporal data with graph neural networks for traffic congestion prediction. In^41^, the author discussed traffic congestion prediction in urban vehicular networks. The article of^42^ discusses predicting traffic congestion of selected routes in Metro Manila. The thesis of^43^ studies the problem of vehicular traffic prediction and congestion avoidance. Lastly, the article of^44^ employed a long short-term memory neural network for traffic congestion prediction.

As our work presented in this article was conducted with traffic data of a street in Jordan, we now turn to related research implemented in the context of Jordan. In^45^, the authors study traffic volume predicting for rural (i.e., intercity) streets in Jordan: the street between Amman and Jerash and the street between Jerash and Irbid. This research examines three predicting methods: linear regression, trend analysis, and empirical Bayesian analysis. The data of this work subsumes traffic volumes for the selected streets from 1996 to 2004 obtained from the Ministry of Public Works and Housing of Jordan. The main objective of this work is to estimate traffic volume to help authorities make rational decisions concerning street network planning and construction.

Another related research done in the context of Jordan is the work of^46^. This work utilizes simulated traffic data for real streets in Jordan to predict the expected level of traffic congestion on the investigated street scenarios. The selected streets of this study are all in Amman: King Abdullah bin Al Hussein II St, Queen Rania Al Abdullah St, and Jordan St. This work’s applied machine learning methods are linear regression, regression tree, and k nearest neighbors regression. The features employed to train machine learning models include the vehicle’s identity, acceleration, angle, distance, lane, position, signals, slope, speed, x-coordinate, and y-coordinate.

## Methodology

### Research Design

**Figure 1:**
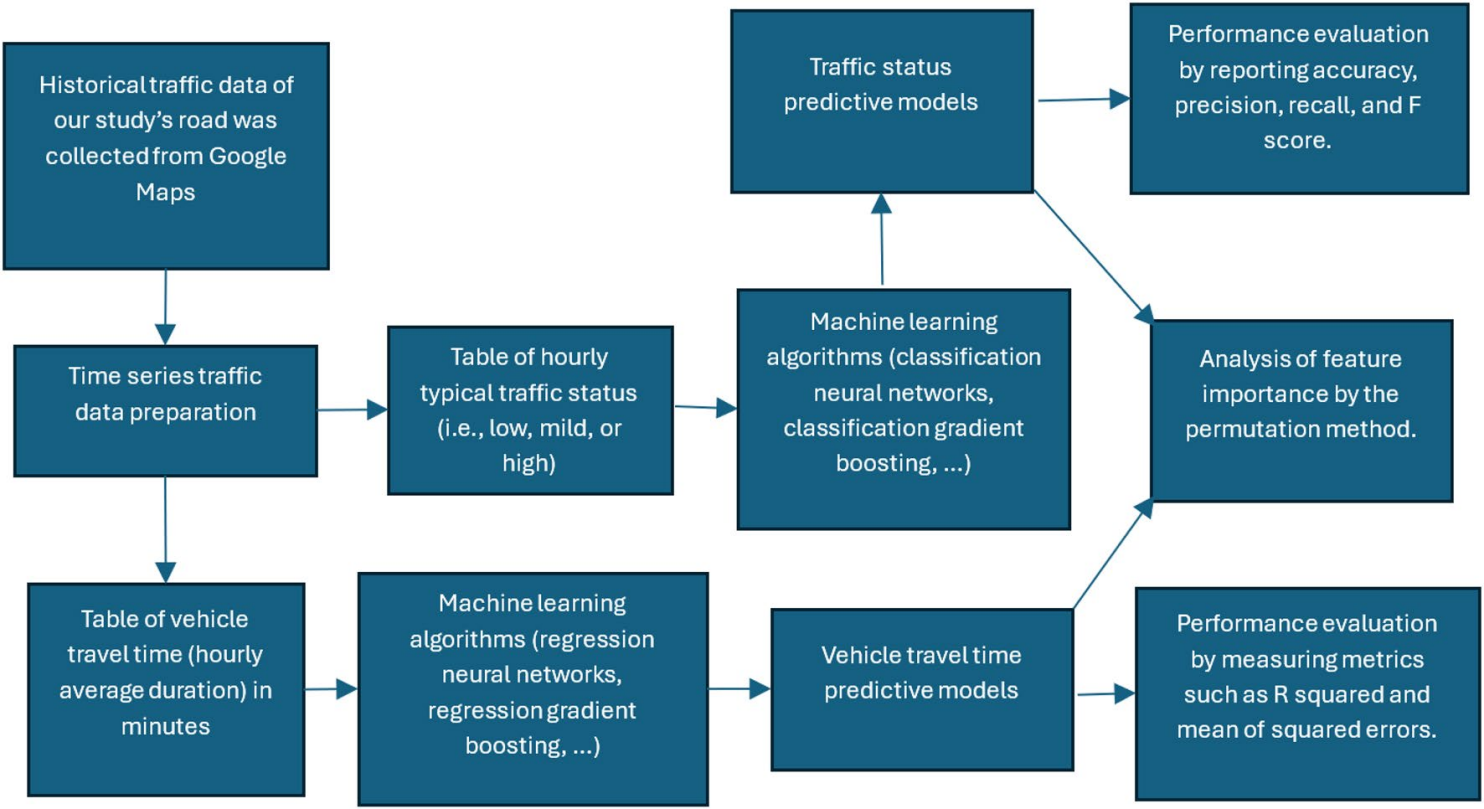
Overview of our research design.

Figure 1 provides an overview of our research design, which involves constructing and analyzing predictive models for vehicle travel time and traffic status on a given street based on historical hourly traffic data. This section elaborates on every stage of the chart in Fig. 1.

### Data description

**Figure 2:**
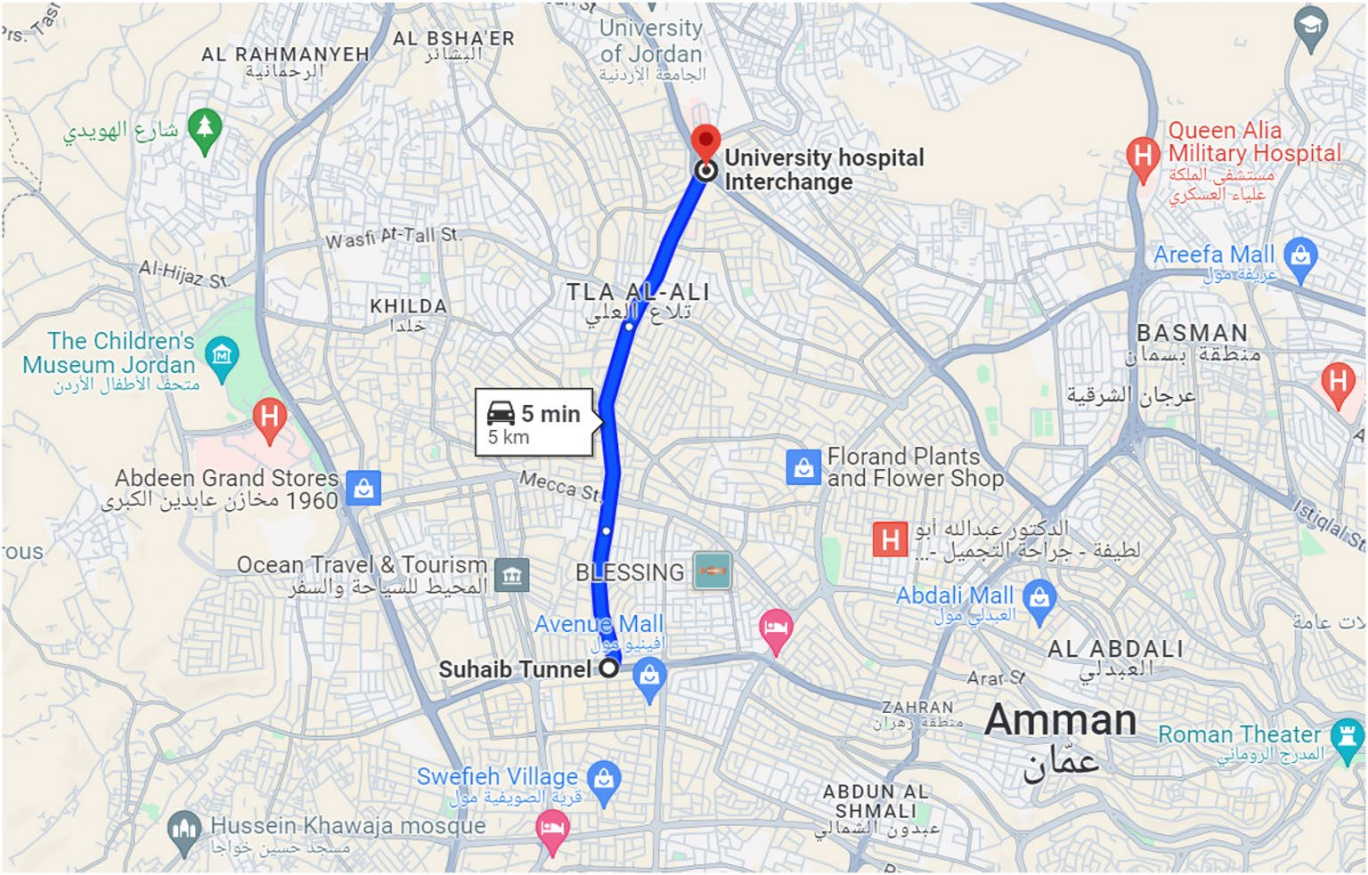
Our study’s street, Al-Madina Al-Monawara St, as viewed in Google Maps.

We obtained historical traffic data from Google Maps for a principal street in the capital city of Jordan, Amman. Amman’s population is about 5 million, whereas Jordan’s is around 11.3 million. Our selected street is Al-Madina Al-Monawara St, which is 5 kilometers long. The street is a two-way street. Our data includes traffic details for one direction of the street, where the data covers the traffic details of the direction from the Suhaib Tunnel to the University Hospital Interchange; see Fig. 2, which views Al-Madina Al-Monawara St.

**Figure 3:**
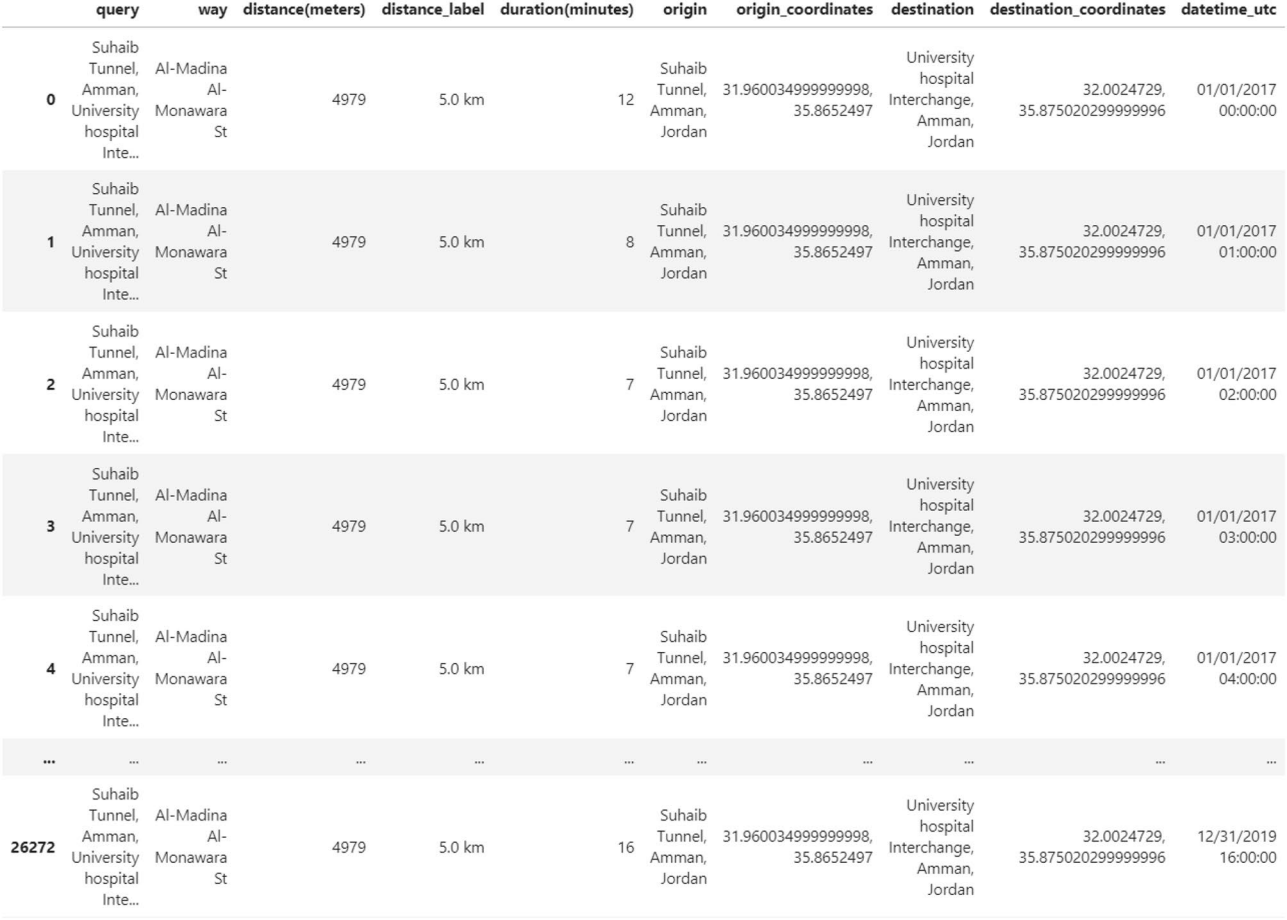
A sample of our data as obtained from Google Maps.

The Google Maps data is a table of hourly traffic data for our study’s street from 1/1/2017 to 31/12/2019. The table includes 26,277 records incorporating two features (among others): date-time (day/month/year hh:mm:ss) and the average vehicle travel time (i.e., average duration in minutes) in the corresponding hour; see Fig. 3, which views a sample of our data.

### Data preparation

From now on, we say “vehicle travel time” instead of “average vehicle travel time” when referring to our historical traffic data. We generated two tables from the obtained Google Maps vehicle travel time data of our study’s street: one table to construct a model to predict the vehicle travel time (after 6 h from a specific point in time) and the other to build a model classifying traffic on the street as low, mild, or high after 6 h from a specific point in time.

**Figure 4:**
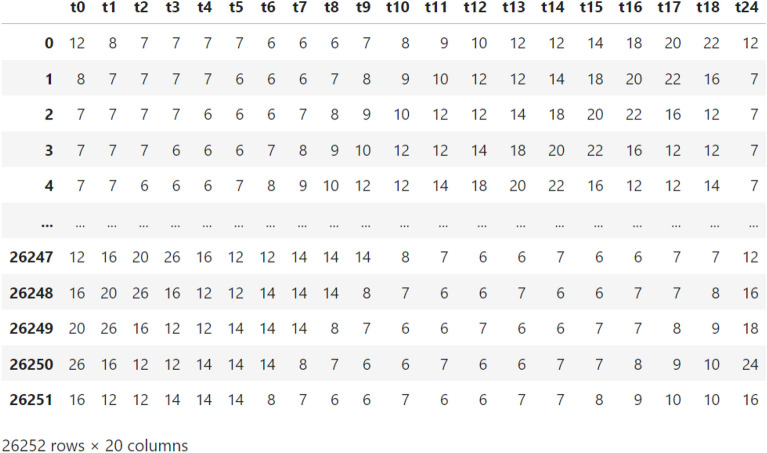
A sample of the time series data prepared for constructing models predicting the vehicle travel time after 6 h on the study’s street.

Hence, to train models for predicting vehicle travel time after 6 h, we prepared a time series table of 26,252 records and 20 columns. Figure 4 shows the prepared time series data, with the input features being the vehicle travel times on the street for consecutive 19 h *t*0–*t*18, and the target feature being the vehicle travel time on the street, *t*24, after 6 h.

**Figure 5:**
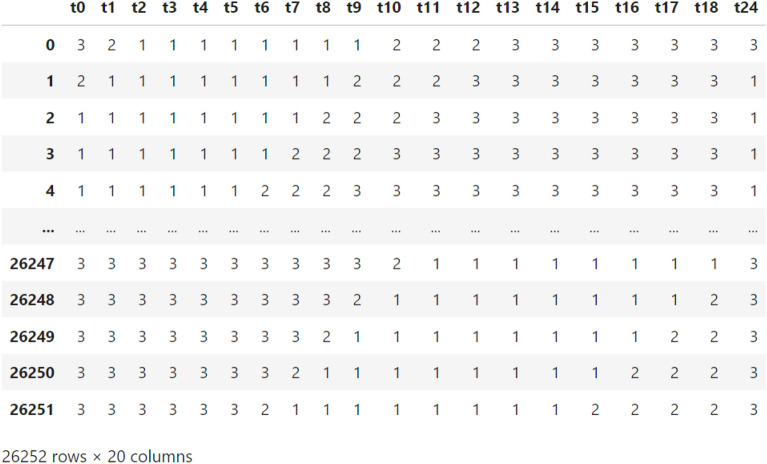
A sample of the time series data prepared for constructing a model predicting the traffic status after 6 h on our study’s street.

Regarding our problem of predicting traffic status (i.e., low, mild, or high), from our time series data (depicted in Fig. 4), we constructed a categorical table of 26,252 rows with 20 columns designating hourly traffic status for consecutive 19 h *t*0–*t*18, where the target feature being the traffic status on the street, *t*24, after 6 h; see Fig. 5. Our traffic status table construction method is as follows: we state 1 (denoting “low traffic”) for a given hour whenever the vehicle travel time in that hour is less than the minimum vehicle travel time in that hour over the three years (2017–2019) plus 2; we state 2 (denoting “mild traffic”) for a given hour if the vehicle travel time in that hour is less than the minimum vehicle travel time in that hour over the three years plus 5; otherwise, we state 3 (denoting “high traffic”). For example, if the vehicle travel time in a given hour (say 12 PM – 1 PM) on 1st Jan 2018 equals 10 minutes, and the minimum travel time on the street in the given hour over the three years is 9 minutes, we note that $10<9+2$, and hence the traffic status on the street is set to “low traffic” for the hour 12 PM–1 PM on 1st Jan 2018.

### Data processing methods

We give an overview of our employed machine learning methods and the reported performance metrics. The interested reader may consult the cited references for further details.

#### Neural networks

Neural networks are a prominent machine learning technique introduced decades ago^47,48^; these days, they are an efficient machine learning technique often utilized in diverse real-world applications. In regression learning, where the output variable is numeric, neural networks learn a nonlinear function $f: {\mathbb {R}}^n \rightarrow {\mathbb {R}}$ from training examples. For binary classification problems, where the output variable can be one of two values, neural networks deduce a nonlinear function $f: {\mathbb {R}}^n \rightarrow \{0,1\}$. A neural network consists of neurons arranged in layers. The first layer of a neural network receives input variables, and the last layer predicts the output variable. One or more hidden layers can exist between the input and output layers. Given a set of *m* training examples $\{\textbf{x}_1,y_1\},\ldots ,\{\textbf{x}_m,y_m\}$ where $\textbf{x}_i$ denotes the values of the input variables $x_1,\ldots ,x_n$ of training example *i* and $y_i$ is the value of the output variable of training example *i*; then, a regression neural network with one-neuron hidden layer infers a function $f(\textbf{x}) = w g(\textbf{w}^T \textbf{x} + b_1)+b_2$ where $\textbf{w} \in {\mathbb {R}}^n$, $w,b_1,b_2 \in {\mathbb {R}}$ are the neural network parameters, *g* is a nonlinear function, called the activation function. Observe, for binary classifications, neural networks learn $f(\textbf{x}) =sigmoid(w g(\textbf{w}^T \textbf{x} + b_1)+b_2)$. For greater details on neural networks, we refer the reader to, e.g.^49,50^.

#### Support vector machines

Support vector machines are a learning method that can be employed for regression and classification problems. Given training vectors $\textbf{x}_i \in {\mathbb {R}}^n$, $i=1,\ldots ,m$, and an output variable vector $\textbf{y} \in {\mathbb {R}}^m$. Then, for regression problems, support vector machines estimate a function $f(\textbf{x})=\textbf{w}^T \phi (\textbf{x}) + b$ by solving the optimization problem

1 $$\begin{aligned} \begin{aligned} \min _{\textbf{w},b,\mathbf {\xi }} \quad&\frac{1}{2}\textbf{w}^{T} \textbf{w} + C \sum _{i=1}^{m}{\xi _{i}}\\ \text {subject to} \quad&y_i(\textbf{w}^T \phi (\textbf{x}_i) +b)\ge 1 - \xi _{i},\\ \quad&\xi _i \ge 0,~i=1,\ldots ,m, \end{aligned} \end{aligned}$$

where $\phi (\textbf{x}_i)$ maps $\textbf{x}_i$ into a higher-dimensional space and $C > 0$ is the regularization parameter. For binary classification problems, support vector machines construct a function $f(\textbf{x})=sign(\textbf{w}^T \phi (\textbf{x}) + b)$. For further details of support vector machines, the reader may consult, e.g.,^51,52^.

#### Nearest neighbors

The idea of the *k*-nearest neighbors algorithm is that the *k* closest training examples in a data set decide the output variable of a query data point. In estimating the output variable of a given query point, the nearest data points can be given uniform weights or assigned different weights according to their distance from the query point. The *k*-nearest neighbors algorithm can be used for regression and classification problems. We refer the reader to^53^ for a complete discussion of the nearest neighbors algorithm.

#### Gradient boosting

Gradient boosting algorithm, introduced in^54^, is an ensemble method that gives a predictive model as a collective of weak prediction models. Gradient boosting constructs an incremental predictive model sequentially; it allows for the optimization of arbitrary differentiable loss functions. In each phase of gradient boosting, a base estimator is fit on the negative gradient of the given loss function being minimized concerning the model values at each training example. Gradient boosting can be applied to solve regression and classification problems. The interested reader may consult^54^ for a fuller presentation of the gradient boosting algorithm.

As gradient boosting is a collective of weak models, we use a collective of decision trees in our study. A decision tree is a widely applied machine learning technique for regression and classification problems; see, e.g.^55^. The aim is to build a tree that predicts the output variable by learning decision rules inferred from training examples. The tree’s internal nodes represent input variables, while the tree’s leaf nodes designate the output variable values. The tree’s branches emitting from an internal node represent the values of the input variable corresponding to the node. A significant benefit of decision trees is that predictions made by decision trees are explainable. In the literature, numerous algorithms for constructing decision trees; see, e.g.^56^.

#### AdaBoost

In AdaBoost, a group of weak learners (e.g., decision trees) are fit on repeatedly modified versions of the data, i.e., the training examples. The weak learners’ predictions are then aggregated through a weighted majority voting. The data modifications at each what-so-called boosting step assign weights to each training example. Initially, all training examples are given equal weight. Then, the weights are individually updated for each successive iteration, and the learning procedure is repeated on the reweighted data. At any iteration, the training examples mispredicted in the previous iteration will receive higher weights in the subsequent learning iteration, allowing the weak learners to pay more attention to those mispredicted instances. For a fuller presentation of AdaBoost, we refer the reader to^57,58^.

### Performance metrics

We evaluated our predictive models using the different prevalent metrics^59^ we now describe. For a data set, let $y_i$ be the true value of the target variable for data point *i*, $\hat{y}_i$ be the prediction of the target variable for data point *i*, and $\bar{y}$ be the mean of all true values of the target variable in the data set, and *n* be the number of training examples in the data set. For the regression models, we measure mean absolute error by

$$\begin{aligned} \frac{\sum _{i=1}^{n} |y_i - \hat{y}_i|}{n},\end{aligned}$$

mean squared error by

$$\begin{aligned} \frac{\sum _{i=1}^{n} (y_i - \hat{y}_i)^2}{n},\end{aligned}$$

median absolute error by

$$\begin{aligned}\text {median}(|y_1 - \hat{y}_1|,\dots ,|y_n - \hat{y}_n|),\end{aligned}$$

$$R^2$$ score by

$$\begin{aligned}1 - \frac{\sum _{i=1}^{n} (y_i - \hat{y}_i)^2}{\sum _{i=1}^{n} (y_i - \bar{y})^2},\end{aligned}$$

explained variance by

$$\begin{aligned} 1 - \frac{var\{y-\hat{y}\}}{var\{y\}},\end{aligned}$$

mean absolute percentage error by

$$\begin{aligned}\frac{1}{n} \sum _{i=1}^{n} \frac{|y_i - \hat{y}_i|}{max\{\epsilon ,|y_i|\}} \text { (where} \epsilon \text {is a tiny positive number)}, \end{aligned}$$

max error by

$$\begin{aligned} \max _i\{|y_i - \hat{y}_i|\}. \end{aligned}$$

For the classification models, we measure the accuracy score by

$$\begin{aligned}\frac{1}{n} \sum _{i=1}^n (\hat{y}_i = y_i).\end{aligned}$$

Further, we measured the standard scores of precision, recall, and *F* measure. Given *n* examples labeled with “positive” or “negative”, the precision of a classifier (concerning the positive label) is equal to the ratio of the number of examples classified as “positive” correctly over the number of all examples that are classified as “positive”; further, the recall of a classifier (concerning the positive label) is equal to the ratio of the number of examples that are classified as “positive” correctly over the number of examples that are indeed “positive”. As a summarization metric combining precision and recall scores, *F* score is the harmonic mean of precision and recall, that is

$$\begin{aligned} F~score = 2 \times \frac{precision \times recall}{precision + recall}.\end{aligned}$$

The Jaccard similarity coefficient with a ground truth label set *y* and predicted label set $\hat{y}$ is defined as the ratio

$$\begin{aligned}\frac{|y \cap \hat{y}|}{|y \cup \hat{y}|}.\end{aligned}$$

Additionally, we analyzed the feature importance of our data based on feature permutation^60^. Permutation-based feature importance is valuable for inspecting models applied to tabular data, mainly when dealing with opaque models like neural networks. By randomly shuffling the value of a single feature, the permutation feature importance measures the resulting reduction in the model’s accuracy score. This process breaks the association between the feature and the target variable, enabling us to gauge how much the model depends on that feature. Next, we give a straightforward narrative of calculating a feature’s importance procedure. Firstly, compute a given model’s reference accuracy score *s*. Then, for each feature *i*, randomly shuffle the data in column *i*. Afterward, compute the corrupted training examples’ accuracy score, $s'$. Thus, the importance of feature *i* equals $s - s'$.

## Experimental results

Recall that we want to predict the vehicle travel time and traffic status (low, mild, or high) after 6 h on our study’s street. Thus, we construct five predictive models using different machine learning algorithms: neural networks, AdaBoost, nearest neighbors, support vector machines, and gradient boosting. In the following subsections, we report our experiments and their results concerning the performance metrics of every constructed predictive model. But before presenting our results, we give a few general comments on the experiments. We created our predictive models using Python 3.9.7 and the machine learning library sklearn 1.2.0^59^. For our regression problems, we utilized the StandardScaler from sklearn.preprocessing such that for each feature *x* of our data, for each value, *d*, of *x*, *d* is replaced by $\frac{d-\mu }{\sigma }$ (i.e., z-score) where $\mu$ is the mean of *x* and $\sigma$ is the standard deviation of *x*. We split our data, 80% for training a model and 20% for testing the model by using sklearn.model_selection.train_test_split. Throughout our experiments, whenever applicable, we ensure the same output across multiple function calls by setting the parameter random_state to an integer.

### Predicting the vehicle travel time after 6 h

#### Our AdaBoost regressor

We used sklearn.ensemble.AdaBoostRegressor to predict the vehicle travel time after 6 h on our study’s street; the base estimator was created from sklearn.tree.DecisionTreeRegressor. Our AdaBoost regressor has a mean absolute error of 0.0046, $R^2$ score of 0.9993, explained variance score of 0.9993, mean squared error of 0.0115, median absolute error of 0.0000, mean absolute percentage error of 0.0005, max error of 3.8290.

**Figure 6:**
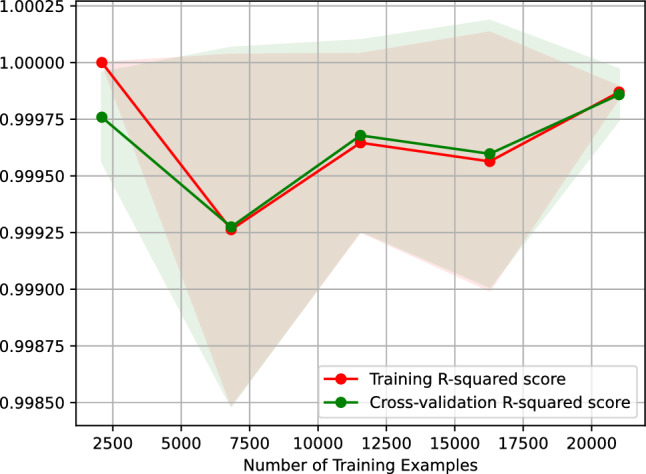
$R^2$ learning curve of our AdaBoost regressor predicting the vehicle travel time after 6 h.

Figure 6 shows the learning curve of our AdaBoost regressor predicting the vehicle travel time after 6 h. Figure 6 plots the $R^2$ score of our AdaBoost regressor against the number of training examples. The learning curve shown in Fig. 6 indicates that the $R^2$ score of predicting the vehicle travel time after 6 h using our AdaBoost regressor remains the same when the regressor is trained on 2500–20,000 examples. Moreover, the figure suggests that our regressor is overfitting-free.

**Figure 7:**
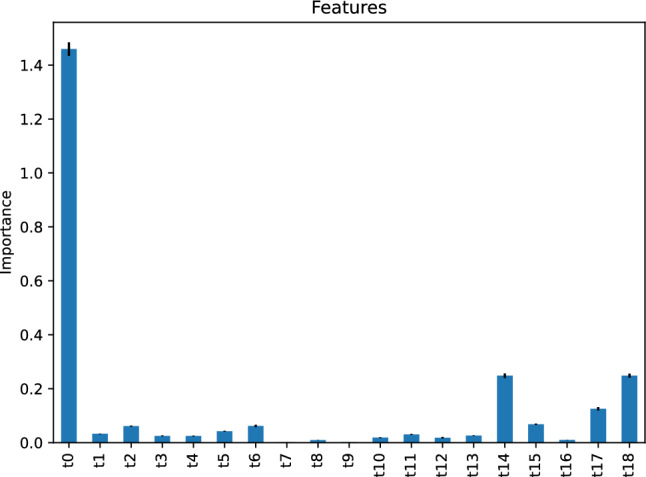
Feature importance of our AdaBoost regressor predicting the vehicle travel time after 6 h.

Figure 7 depicts the feature importance of our AdaBoost regressor, indicating that the most critical feature of our AdaBoost model predicting the vehicle travel time after 6 h is the vehicle travel time, *t*0, 24 h before the target hour. The second important features are *t*14 and *t*18, the vehicle travel time 6 h and 10 h before the target hour, respectively.

#### Our neural network regressor

We used sklearn.neural_network.MLPRegressor to build our neural network regressor with two hidden layers and 200 neurons in each layer for predicting the vehicle travel time after 6 h on our study’s street. Our neural network regressor has a mean absolute error of 0.0612, $R^2$ score of 0.9994, explained variance score of 0.9994, mean squared error of 0.0101, median absolute error of 0.0610, mean absolute percentage error of 0.0067, max error of 3.9016.

**Figure 8:**
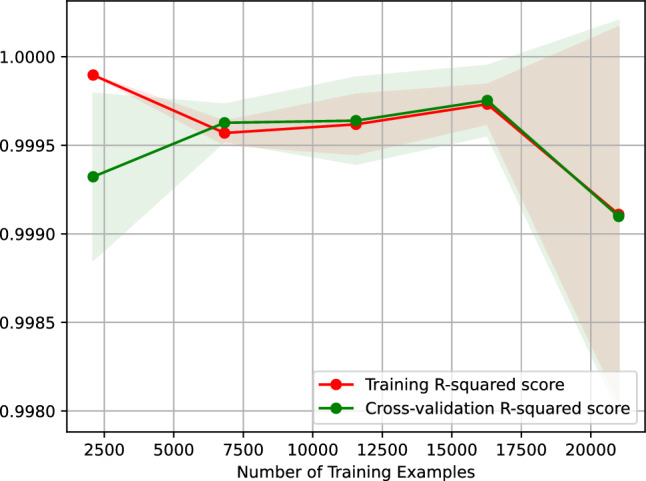
$R^2$ learning curve of our neural network regressor predicting the vehicle travel time after 6 h.

Figure 8 shows the learning curve of our neural network regressor predicting the vehicle travel time after 6 h. Figure 8 plots the $R^2$ score of our neural network regressor against the number of training examples. The learning curve shown in Fig. 8 indicates that the $R^2$ score of predicting the vehicle travel time after 6 h using our neural network regressor remains the same when the regressor is trained on 2500–20,000 examples. On top of that, the figure suggests that our regressor is overfitting-free.

**Figure 9:**
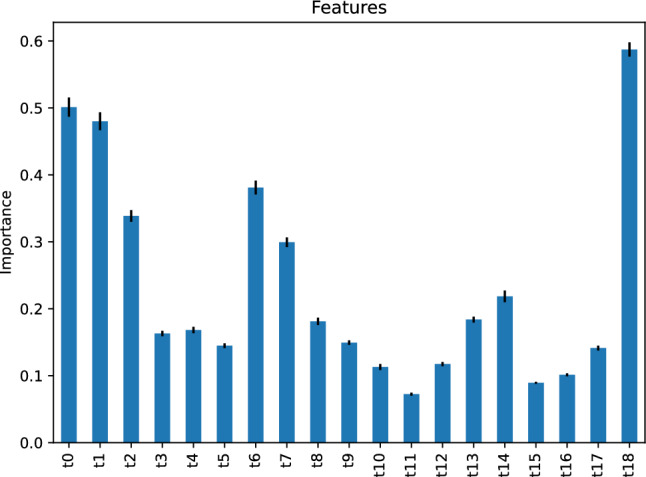
Feature importance of our neural network regressor predicting the vehicle travel time after 6 h.

Figure 9 depicts the importance of our neural network regressor’s features, suggesting that the most critical feature for predicting the vehicle travel time after 6 h on our study’s street is the vehicle travel time, *t*18, 6 h before the target hour. The second most important features are *t*0 and *t*1, the vehicle travel time 24 h and 23 h before the target hour, respectively.

#### Our gradient boosting regressor

We exploited sklearn.ensemble.GradientBoostingRegressor with a learning rate of 0.5 to create our gradient boosting regressor for predicting the vehicle travel time after 6 h on our study’s street. Our gradient boosting regressor has a mean absolute error of 0.0337, $R^2$ score of 0.9996, explained variance score of 0.9996, mean squared error of 0.0056, median absolute error of 0.0222, mean absolute percentage error of 0.0035, max error of 3.8984.

**Figure 10:**
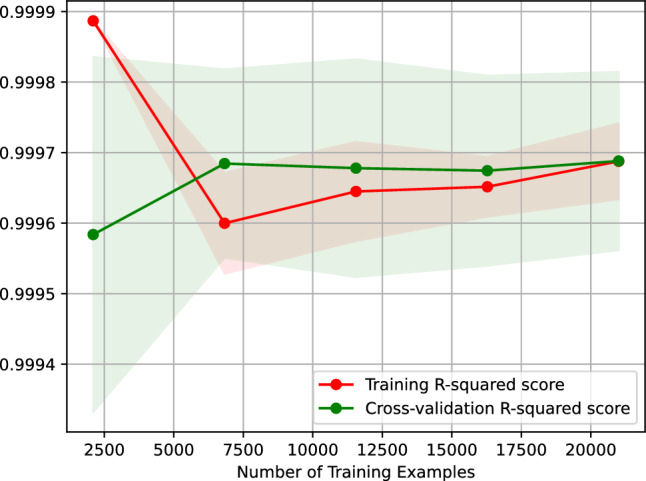
$R^2$ learning curve of our gradient boosting regressor predicting the vehicle travel time after 6 h.

Figure 10 shows the learning curve of our gradient boosting regressor predicting the vehicle travel time after 6 h. Figure 10 plots the $R^2$ score of our gradient boosting regressor against the number of training examples. The learning curve of Fig. 10 suggests that the $R^2$ score of predicting the vehicle travel time after 6 h using our gradient boosting regressor remains the same when the regressor is trained on 2500–20,000 examples. Besides, the figure implies that our regressor is overfitting-free.

**Figure 11:**
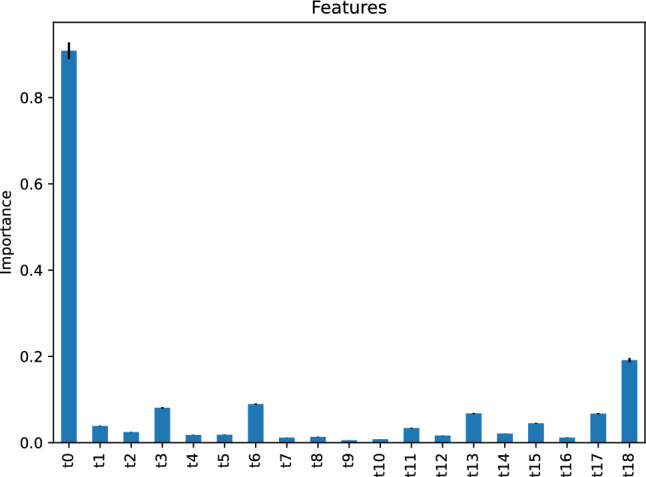
Feature importance of our gradient boosting regressor predicting the vehicle travel time after 6 h.

Figure 11 depicts the feature importance of our gradient boosting regressor. It indicates that the most critical feature for predicting the vehicle travel time after 6 h is the vehicle travel time, *t*0, 24 h before the target hour. The second most important feature is the vehicle travel time, *t*18, 6 h before the target hour.

#### Our nearest neighbors regressor

We used sklearn.neighbors.KNeighborsRegressor to create our nearest neighbors regressor for predicting the vehicle travel time after 6 h on our study’s street. Our nearest neighbors regressor has a mean absolute error of 0.0007, $R^2$ score of 0.9998, explained variance score of 0.9998, mean squared error of 0.0030, median absolute error of 0.0000, mean absolute percentage error of 0.0000, max error of 4.000.

**Figure 12:**
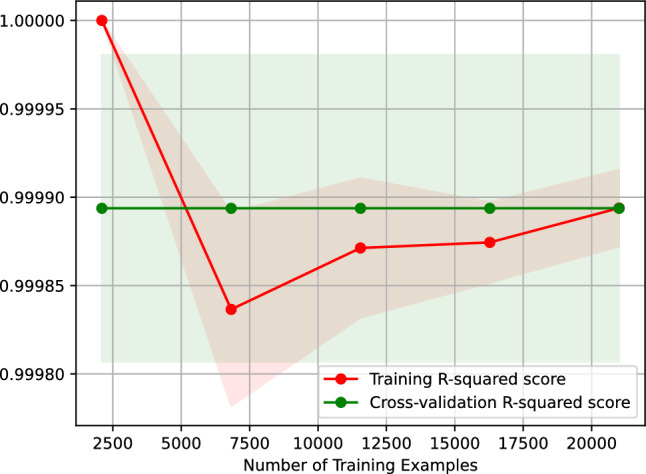
$R^2$ learning curve of our nearest neighbors regressor predicting the vehicle travel time after 6 h.

Figure 12 shows the learning curve of our nearest neighbors regressor predicting the vehicle travel time after 6 h. Figure 12 plots the $R^2$ score of our nearest neighbors regressor against the number of training examples. The learning curve of Fig. 12 suggests that the $R^2$ score of predicting the vehicle travel time after 6 h using our nearest neighbors regressor remains the same when the regressor is trained on 2500–20,000 examples. Further, the figure implies that our regressor is overfitting-free.

**Figure 13:**
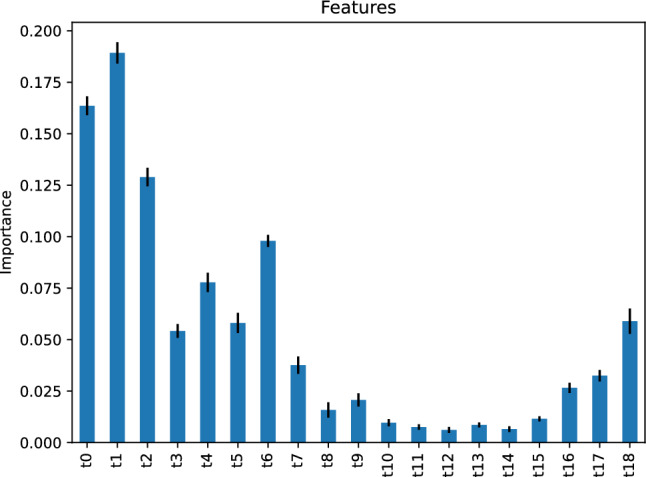
Feature importance of our nearest neighbors regressor predicting the vehicle travel time after 6 h.

Figure 13 depicts the feature importance of our nearest neighbor regressor. It indicates that the most critical feature of our nearest neighbor regressor predicting the vehicle travel time after 6 h is the vehicle travel time, *t*1, 23 h before the target hour. The second most important feature is *t*0, the vehicle travel time 24 h before the target hour.

#### Our support vector machine regressor

We exploited sklearn.svm.SVR to create our support vector machine regressor for predicting the vehicle travel time after 6 h on our study’s street. Our support vector machine regressor has a mean absolute error of 0.1817, $R^2$ score of 0.9898, explained variance score of 0.9898, mean squared error of 0.1770, median absolute error of 0.1000, mean absolute percentage error of 0.0166, max error of 4.7504.

**Figure 14:**
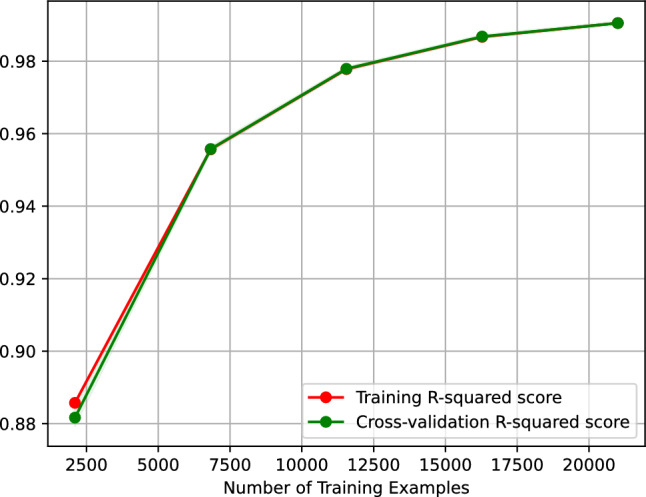
$R^2$ learning curve of our support vector machine regressor predicting the vehicle travel time after 6 h.

Figure 14 shows the learning curve of our support vector machine regressor predicting the vehicle travel time after 6 h. It plots the $R^2$ score of our support vector machine regressor against the number of training examples. The learning curve of Fig. 14 indicates that the $R^2$ score of predicting the vehicle travel time after 6 h using our support vector machine regressor improves as the number of training examples increases. Equally, the figure implies that our regressor is overfitting-free.

**Figure 15:**
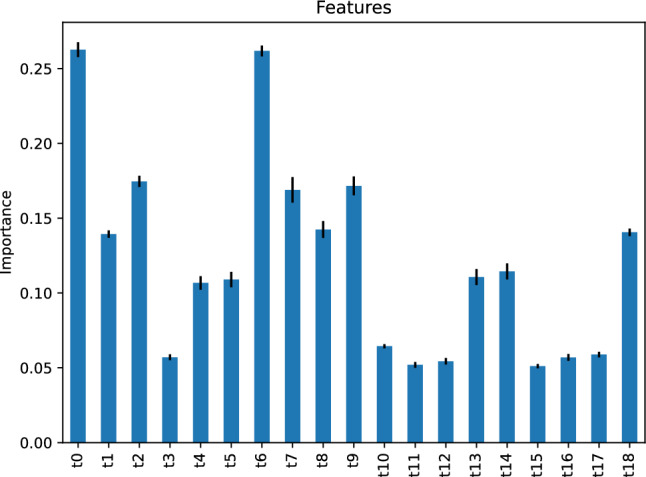
Feature importance of our support vector machine regressor predicting the vehicle travel time after 6 h.

Figure 15 depicts the feature importance of our support vector machine regressor, implying that the most critical features of our support vector machine regressor predicting the vehicle travel time after 6 h are *t*0 and *t*6, respectively, 24 h and 19 h before the target hour. The second most important features are *t*2, *t*7, and *t*9, respectively, the vehicle travel time 22 h, 17 h, and 15 h before the target hour.

### Predicting the traffic status after 6 h

#### Our AdaBoost classifier

**Figure 16:**
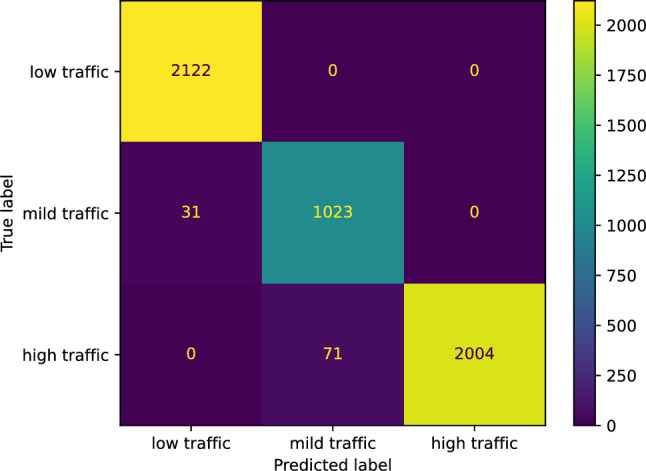
The confusion matrix of our AdaBoost classifier predicting the traffic status after 6 h.

We used sklearn.ensemble.AdaBoostClassifier to create our AdaBoost classifier for predicting the traffic status after 6 h on our study’s street; the base estimator was created as DecisionTreeClassifier from the sklearn.tree. Our AdaBoost classifier achieved an accuracy of 0.9805. We now report the other performance scores our AdaBoost classifier reached for the three classes: low, mild, and high traffic, respectively. The *F* score of our AdaBoost classifier is [0.9927 0.9525 0.9825]. The precision score is [0.9856 0.9351 1.0000]. The recall score is [1.0000 0.9705 0.9657], and the Jaccard score is [0.9856 0.9093 0.9657]. Figure 16 presents the confusion matrix of our AdaBoost classifier.

**Figure 17:**
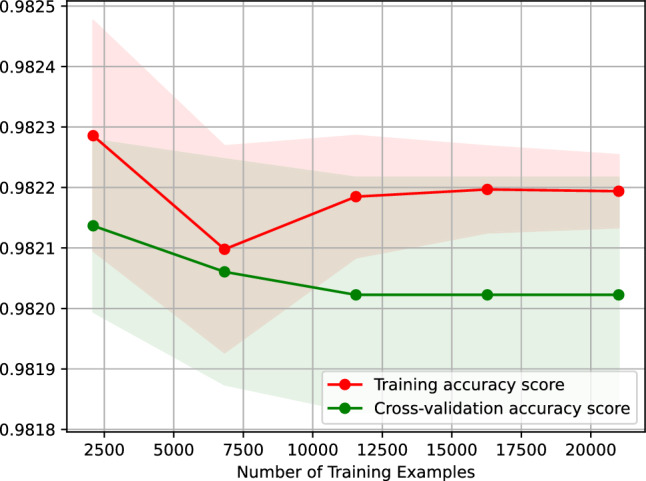
Accuracy-score learning curve of our AdaBoost classifier predicting the traffic status after 6 h.

Figure 17 shows the learning curve of our AdaBoost classifier predicting the traffic status after 6 h. Figure 17 plots the accuracy score of our AdaBoost classifier against the number of training examples. The learning curve illustrated in Fig. 17 indicates that the accuracy of predicting the traffic status after 6 h using our AdaBoost classifier remains the same when the classifier is trained on 2500–20,000 examples. Likewise, Fig. 17 indicates that our AdaBoost classifier is overfitting-free.

**Figure 18:**
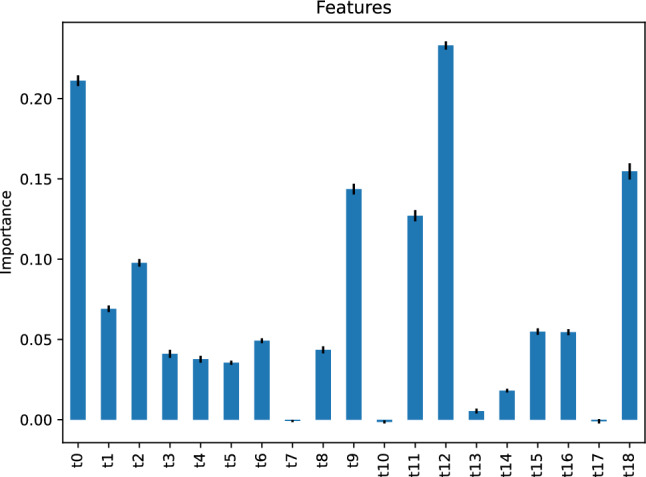
Feature importance of our AdaBoost classifier predicting the traffic status after 6 h.

Figure 18 demonstrates the importance of the AdaBoost classifier’s features. The figure implies that the most critical feature of our AdaBoost classifier for predicting the traffic status after 6 h is the traffic status *t*12, 12 h before the target hour. The second-most important feature is the traffic status *t*0, 24 h before the target hour.

#### Our neural network classifier

**Figure 19:**
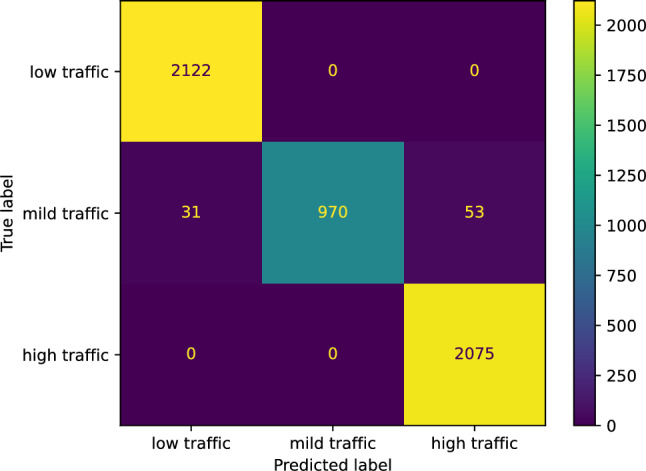
The confusion matrix of our neural network classifier predicting the traffic status after 6 h.

We employed sklearn.neural_network.MLPClassifier to build our neural network classifier with two hidden layers and 200 neurons in each layer for predicting the traffic status after 6 h on our study’s street. Our neural network classifier achieved an accuracy of 0.9840. We now state the other performance scores our neural network classifier reached for the three classes: low, mild, and high traffic, respectively. The *F* score of our neural network classifier is [0.9927 0.9584 0.9873]. The precision score is [0.9856 1.0000 0.9750]. The recall score is [1.0000 0.9203 1.0000], and the Jaccard score is [0.9856 0.9203 0.9750]. Figure 19 shows the confusion matrix of our neural network classifier predicting the traffic status after 6 h.

**Figure 20:**
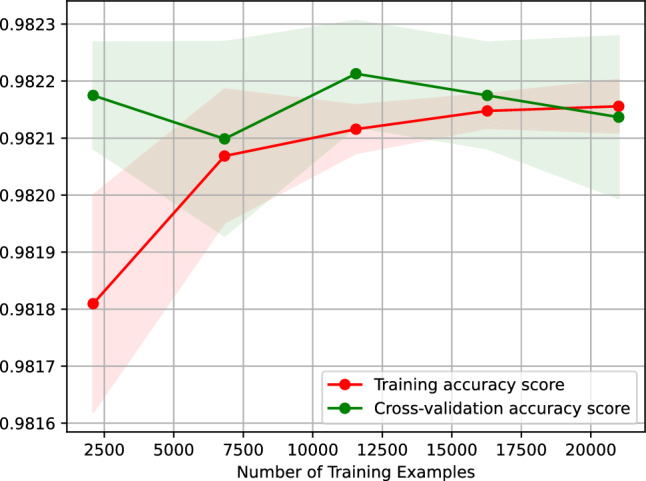
Accuracy-score learning curve of our neural network classifier predicting the traffic status after 6 h.

Figure 20 presents the learning curve of our neural network classifier for predicting the traffic status after 6 h. Figure 20 plots the accuracy score of our neural network classifier against the number of training examples. The learning curve shown in Figure 20 indicates that the accuracy of predicting the traffic status after 6 h using our neural network classifier remains the same when trained on 2,500-20,000 examples. Besides, the figure suggests that our neural network classifier is overfitting-free.

**Figure 21:**
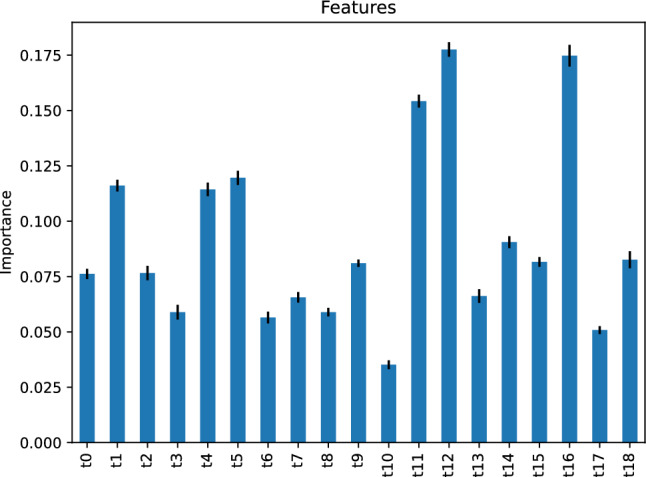
Feature importance of our neural network classifier predicting the traffic status after 6 h.

Figure 21 graphs the neural network classifier’s feature importance, implying that our two most critical features for predicting the traffic status after 6 h are *t*12 and *t*16, respectively, the traffic status 12 and 8 h before the target hour.

#### Our gradient boosting classifier

**Figure 22:**
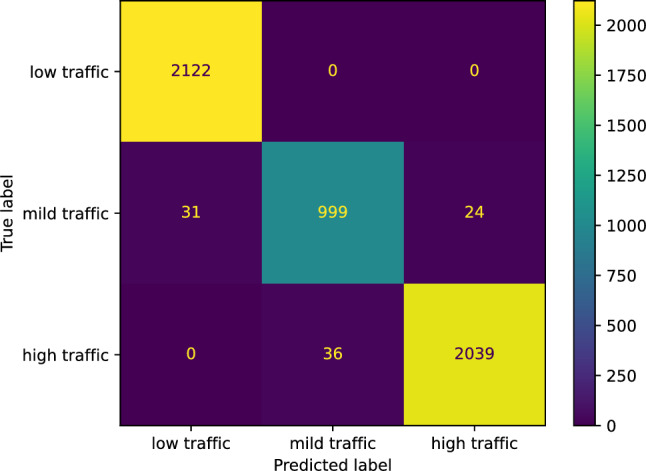
The confusion matrix of our gradient boosting classifier predicting the traffic status after 6 h.

We used sklearn.ensemble.GradientBoostingClassifier to create our gradient boosting classifier for predicting the traffic status after 6 h. Our gradient boosting classifier achieved an accuracy score of 0.9826. We now list the other performance scores our gradient boosting classifier reached for the three classes: low, mild, and high traffic, respectively. The *F* score of our gradient boosting classifier is [0.9927 0.9564 0.9855]; the precision score is [0.9856 0.9652 0.9883]; the recall score is [1.0000 0.9478 0.9826]; the Jaccard score is [0.9856 0.9165 0.9714]. Figure 22 shows the confusion matrix of our gradient boosting classifier.

**Figure 23:**
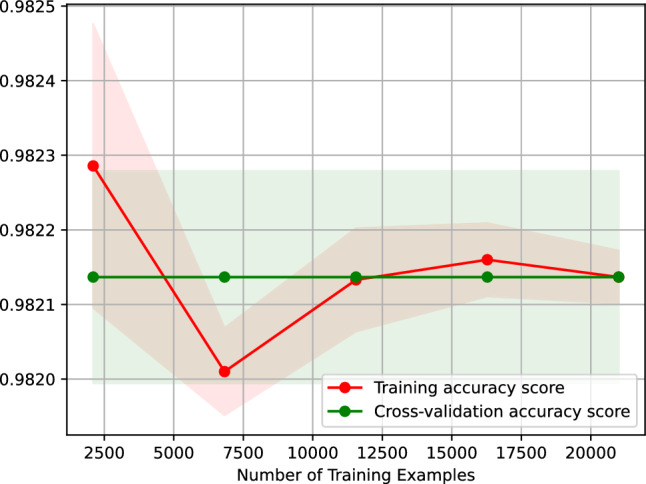
Accuracy-score learning curve of our gradient boosting classifier predicting the traffic status after 6 h.

Figure 23 shows the learning curve of our gradient boosting classifier predicting the traffic status after 6 h. Figure 23 plots the accuracy score of our gradient boosting classifier against the number of training examples. The learning curve shown in Figure 23 suggests that the accuracy of predicting the traffic status after 6 h using our gradient boosting classifier remains the same when trained on 25,500–20,000 examples. Additionally, the figure suggests that our gradient boosting classifier is overfitting-free.

**Figure 24:**
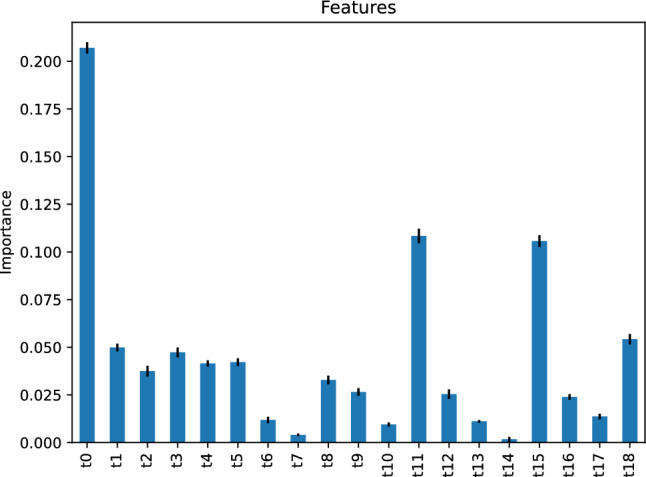
Feature importance of our gradient boosting classifier predicting the traffic status after 6 h.

The feature importance of our gradient boosting classifier is depicted in Fig. 24, implying that the most critical feature of our gradient boosting classifier predicting the traffic status after 6 h is the traffic status, *t*0, 24 h before the target hour. The second most important features are *t*11 and *t*15, respectively, the traffic status 13 and 9 h before the target hour.

#### Our nearest neighbors classifier

**Figure 25:**
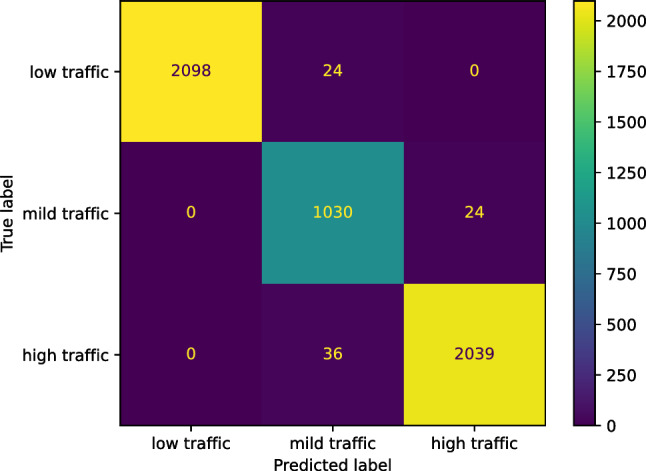
The confusion matrix of our nearest neighbors classifier predicting the traffic status after 6 h.

We utilized sklearn.neighbors.KNeighborsClassifier to construct our nearest neighbors classifier for predicting the traffic status after 6 h on our study’s street. The classifier achieved an accuracy of 0.9840. We now report the other performance scores our nearest neighbor classifier reached concerning the three classes: low, mild, and high traffic, respectively. The *F* score of our classifier is [0.9943 0.9608 0.9855]; the precision score is [1.0000 0.9449 0.9883]; the recall score is [0.9886 0.9772 0.9826]; the Jaccard score is [0.9886 0.9245 0.9714]. Figure 25 illustrates the confusion matrix of our nearest neighbors classifier.

**Figure 26:**
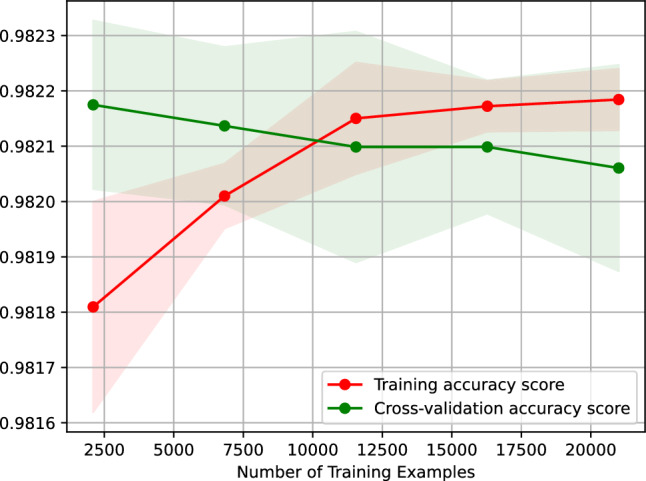
Accuracy-score learning curve of our nearest neighbors classifier predicting the traffic status after 6 h.

Figure 26 shows the learning curve of our nearest neighbors classifier predicting the traffic status after 6 h. Figure 26 graphs the accuracy score of our nearest neighbors classifier against the number of training examples. The learning curve shown in Figure 26 suggests that the accuracy of predicting the traffic status after 6 h using our nearest neighbors classifier remains the same when the classifier is trained on 2500–20,000 examples. Moreover, the figure indicates that our nearest neighbors classifier is overfitting-free.

**Figure 27:**
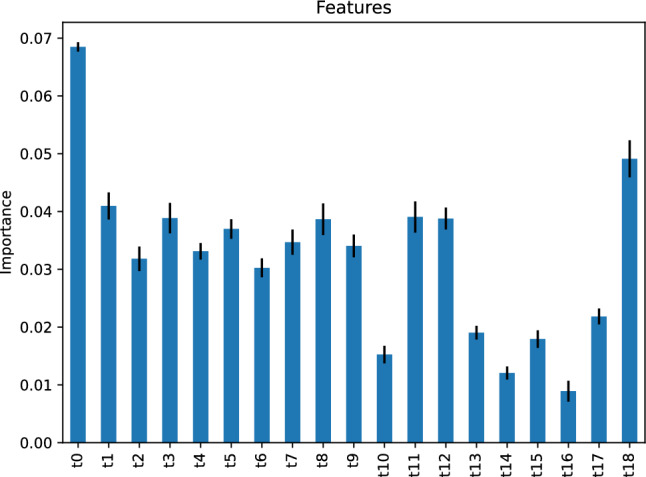
Feature importance of our nearest neighbors classifier predicting the traffic status after 6 h.

Figure 27 depicts the feature importance of our nearest neighbors classifier. It indicates that the most significant feature for predicting the traffic status after 6 h is the traffic status, *t*0, 24 h before the target hour. The second most important feature is *t*18, the traffic status 6 h before the target hour.

#### Our support vector machine classifier

**Figure 28:**
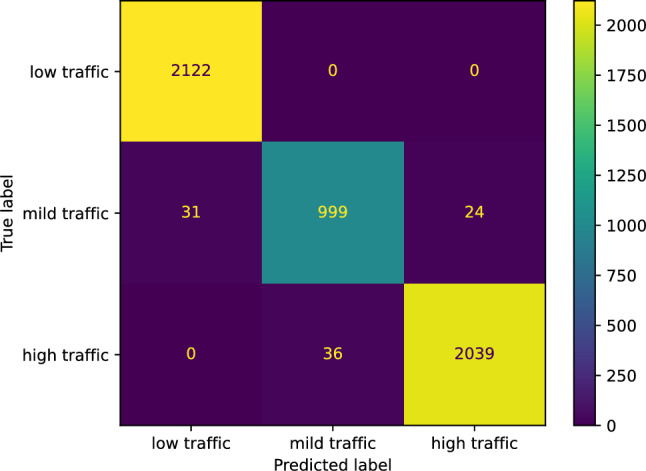
The confusion matrix of our support vector machine classifier predicting the traffic status after 6 h.

We utilized sklearn.svm.SVM to construct our support vector machine classifier for predicting the traffic status after 6 h on our study’s street. Our support vector machine classifier achieved an accuracy of 0.9826. We now state the other performance scores our support vector machine classifier reached concerning the three classes: low, mild, and high traffic, respectively. The *F* score of our classifier is [0.9927 0.9564 0.9855]; the precision score is [0.9856 0.9652 0.9883]; the recall score is [1.0000 0.9478 0.9826]; the Jaccard score is [0.9856 0.9165 0.9714]. Figure 28 shows the confusion matrix of our support vector machine classifier.

**Figure 29:**
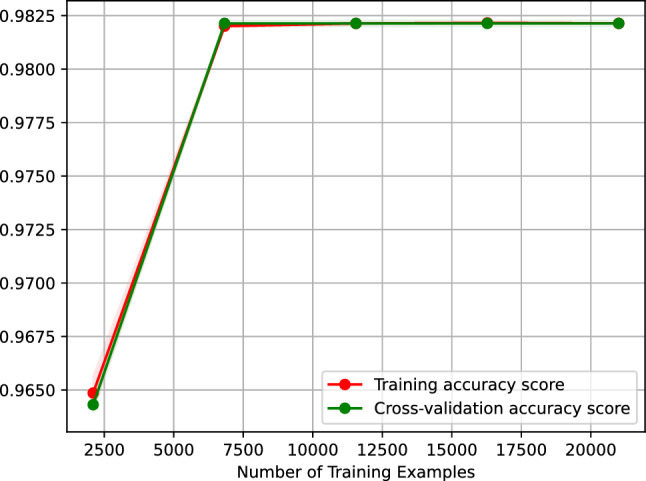
Accuracy-score learning curve of our support vector machine classifier predicting the traffic status after 6 h.

Figure 29 shows the learning curve of our support vector machine classifier, which predicts the traffic status after 6 h. Figure 29 traces the accuracy score of our nearest neighbors classifier against the number of training examples. The learning curve shown in Fig. 29 implies that the accuracy of predicting the traffic status after 6 h using our support vector machine classifier remains the same when the classifier is trained on 7000–20,000 examples. Moreover, the figure suggests that our support vector machine classifier is overfitting-free.

**Figure 30:**
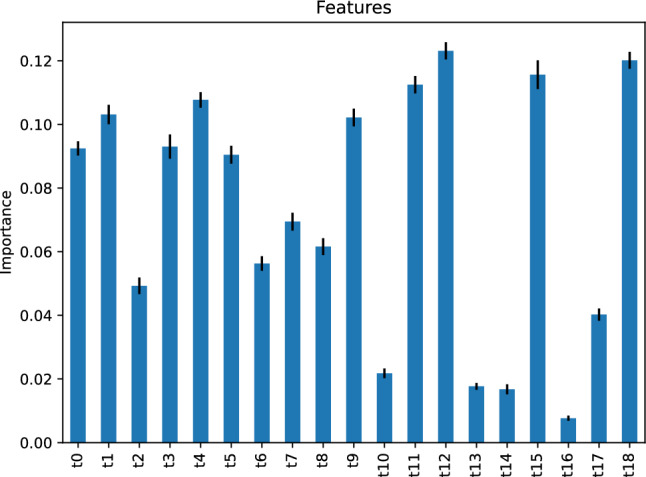
Feature importance of our support vector machine classifier predicting the traffic status after 6 h.

Figure 30 depicts the feature importance of our support vector machine classifier, stating that several features are critical to predicting the traffic status after 6 h using our support vector machine classifier; most notably, the feature *t*12, the traffic status 12 h before the target hour.

### Discussions

#### Summary of results

Table 1 compares the performance of our models in predicting the vehicle travel time after 6 h on our study’s street. We note that our models’ performance is comparable without significant variations. However, the models’s most important predictors vary greatly, as observed in the last two rows in Table 1; recall that $t_i$ refers to the average vehicle travel time during the hour *i*.

**Table 1:** Summary of results of models predicting the vehicle travel time after 6 h on our study’s street.

	AdaBoost	Neural net.	Gradient boos.	Nearest neighbors	Support vect. mach.
Mean absolute error	0.0046	0.0612	0.0337	0.0007	0.1817
R squared score	0.9993	0.9994	0.9996	0.9998	0.9898
Explained variance score	0.9993	0.9994	0.9996	0.9998	0.9898
Mean squared error	0.0115	0.0101	0.0056	0.0030	0.1770
Median absolute error	0.0000	0.0610	0.0222	0.0000	0.1000
Mean abs. percent. error	0.0005	0.0067	0.0035	0.0000	0.0166
Max error	3.8290	3.9016	3.8984	4.0000	4.7504
First important predictor	*t*0	*t*18	*t*0	*t*1	*t*0 & *t*6
Second important predictor	*t*14 & *t*18	*t*0 & *t*1	*t*18	*t*0	*t*2, *t*7, & *t*9

Table 2 compares the performance of our models in predicting the traffic status after 6 h on our study’s street. We note that our models’ performance is comparable without significant variations. However, the models’s most important predictors vary greatly, as observed in the last two rows in Table 2.

**Table 2:** Summary of results of models predicting the traffic status after 6 h on our study’s street.

	AdaBoost	Neural net.	Gradient boost.	Nearest neighbors	Support vect. mach.
Accuracy score	0.9805	0.9840	0.9826	0.9840	0.9826
F score (low traffic)	0.9927	0.9927	0.9927	0.9943	0.9927
F score (mild traffic)	0.9525	0.9584	0.9564	0.9608	0.9564
F score (high traffic)	0.9825	0.9873	0.9855	0.9855	0.9855
Jaccard score (low traffic)	0.9856	0.9856	0.9856	0.9886	0.9856
Jaccard score (mild traffic)	0.9093	0.9203	0.9165	0.9245	0.9165
Jaccard score (high traffic)	0.9657	0.9750	0.9714	0.9714	0.9714
First important predictor	*t*12	*t*12 & *t*16	*t*0	*t*0	*t*12
Second important predictor	*t*0	*t*11	*t*11 & *t*5	*t*18	*t*18

#### Implication of results

We carried out the experiments reported earlier to test our two hypotheses. Regarding our first hypothesis, we wanted to check if the vehicle travel time after 6 h on a city street can be predicted to a certain degree, provided the hourly vehicle travel time on the given street in the last 19 h. For our second hypothesis, we wanted to see if the traffic status (as low, mild, or high traffic) on a city street can be predicted, provided the hourly traffic status on the given street in the last 19 h. We examined our hypotheses on a main street in the capital city of Jordan, Amman. Our experimental results showed that our created predictive models are highly accurate, with an accuracy of around 98–99%. Thus, our results positively answer the questions implied by our study’s hypothesis. This is because our predictive models are highly accurate concerning the prediction tasks entailed by our hypothesis. By utilizing the hourly vehicle travel time on our study’s street in the last 19 h, our models show high accuracy in predicting the vehicle travel time after 6 h on the street. Likewise, by employing the hourly traffic status on the street in the last 19 h, our models show high accuracy in predicting the traffic status after 6 h on the street. Concerning our investigations for the most critical predictors of vehicle travel time and traffic status after 6 h on the street, the variation between our predictive models is notable.

#### Limitation of results

We note that the limitation of our study is that our hypotheses are examined on one street. To strengthen our results, our experiments can be replicated (perhaps in other cities or countries) on streets with different characteristics such as street length, street width, number of traffic lights on the street, number of junctions on the street, and number of shops on the street. Also, our predictive models were constructed based entirely on hourly traffic status (and vehicle travel time) on the concerned street in the last 19 h. Still, other possible predictors, such as weather conditions, special events, unplanned holidays, construction works, accidents, and emergent street maintenance, were overlooked by our examinations because such data features were not available in this study.

## Conclusion

We reported our experimental study of testing the hypothesis of whether the vehicle travel time (respectively, the traffic status) after 6 h on a given street can efficiently be predicted based on the hourly vehicle travel time (respectively, the traffic status) on the street in the last 19 h. As our findings positively confirm the questions of our hypothesis, our study impacts how city map applications estimate the vehicle travel time on a given street. Although map applications are excellent in tracking the instant traffic status of streets, map applications (e.g., Google Maps) give users a loose estimate if the users query about vehicle travel time for a trip that will start later after a while, say after 6 h. Therefore, for personal planning ahead of city trips, our results encourage map applications to incorporate predictive models into their systems, providing their users with more effective tools for navigating city streets. On the other hand, for authorities responsible for street traffic management, our results are encouraging to exploit street traffic data in the last few hours to locate streets with traffic congestion, and thus, traffic authorities are more productive in controlling traffic jams on the identified streets.

In the future, we aim to investigate the possibility of predicting the vehicle travel time (and traffic status) on a given street for the next day to enable the concerned users to plan and have plenty of time to take any required actions. Take the scenario where very critical events need to be arranged at the earliest for either personal purposes, such as a wedding event, or for street traffic management purposes, dealing with a football match, for instance, where traffic authorities might be more interested in identifying streets with low traffic to set an effective street diversion plan to get most of the traffic away from the location of the event.

## Data Availability

The dataset used in the current study is available from the author upon reasonable request.
